# Research and Remedies

**Published:** 1912-07-13

**Authors:** 


### RESEARCH AND REMEDIES.

### Chemical Sterilisation of Drinking Water.

For some years Captain Nesfield, of the Indian Medical Service, has been an advocate of the chemical method of rendering water supplies fit for use by troops in the field or on manœuvres. In the *Journal of the Royal Army Medical Corps* he gives a full account of his system, and defends himself against the charge of having plagiarised it from a French source. He uses three tablets, A, B, and C. A consists of potassium iodide and iodate mixed together in the molecular proportions of five to one; B consists of citric or tartaric acid; C is hyposulphite of soda. One and a half grains of each tablet is required to deal with one gallon of water. A and B are dissolved together in the least possible quantity of water—about ten drops. The resulting solution and precipitate are then added to the full bulk of water and stirred up. The water acquires a faint yellow tinge, and is allowed to stand for five minutes. Tablet C is then dissolved in a small quantity of the tinted water and added to the main bulk, which is once more stirred. The water is then tasteless and fit to drink; it contains one part of iodine in 70,000. Muddy water should first be strained through cotton wool. Water known to be grossly contaminated may need double the quantity of tablets; and on the other hand water known to be pretty good can safely be used with only half the usual addition. Indian regiments have consumed water thus sterilised for three years on end without harm; and one corps has done so for seven years. Everyone who has practical experience of the difficulty of getting boiled water in the field and of keeping filter carts in working order must wish to see Captain Nesfield's tablets scientifically tried and tested for the British Army.

### A Research on Radium.

Although the Radium Institute has been established for some time, the amount of exact information upon the modes of action of this mysterious product is still very small. Dr. Bellingham Smith publishes in the *Quarterly Journal of Medicine* a list of findings upon the excretion of radium; although somewhat indefinite on many points, they nevertheless form an advance upon current knowledge. After the administration, by the mouth or by injection, of radium a widespread degree of radio-activity is evident throughout the body. Elimination of radium takes place principally and rapidly by the bowel, in a minor and slower degree by the kidneys, and (in mice at least) not at all through the liver or skin. Radium *emanations* can be obtained in solution in various media, and can be introduced into the body in small doses by inhalation, feeding, or injection. After any such administration, however conducted, a general radio-activity of very brief duration is caused throughout the body. Elimination of the emanations, in contrast to that of radium itself, takes place almost entirely through the lungs, and to a very slight extent through the kidneys. If insoluble salts of radium are administered by the mouth little if any will be absorbed; but if the same preparations are given by injection into the tissues, elimination is so slow that they may be regarded as permanently present.

### The Microbe of Appendicitis.

Some ten years ago Drs. Poynton and Paine announced the discovery of the causal organism of acute rheumatism, a diplococcus to which they gave the name of *d. rheumaticus*. Since then they have repeatedly upheld the specific nature of this organism, and other investigators have agreed with them; but bacteriologists as a whole have not accepted the position of this organism as proved beyond dispute. The same observers have lately, in continuation of their researches, concluded that the diplococcus rheumaticus may be, and often is, the origin of acute appendicitis in childhood. They regard an attack of this disease as the "result of a blood infection determining to this organ rather than to some local hurt or infection." Thus they inoculated twenty-five rabbits intravenously with a culture of the micrococcus. One developed pericarditis, twenty-four got arthritis, and four had acute appendicitis. The infection, it is supposed, occurs from the submucosa, owing to necrosis of lymphoid tissue there and destruction of Lieberkuhn's follicles. The offending organism was recovered from the lesions, which occurred in the middle third of the appendix, the commonest site also of appendicitis in man. Dr. Poynton, who contributes an account of his views to the *Medical Chronicle*, admits that caution is necessary in applying to mankind the results of experimental work on animals, and also that up to a recent date he had seen no reason clinically for associating appendicitis and rheumatism.

